# Seeing red: Light- and temperature-dependent complex formation reveal new insights into phytochrome activity

**DOI:** 10.1093/plcell/koae255

**Published:** 2024-09-20

**Authors:** Rory Osborne

**Affiliations:** Assistant Features Editor, The Plant Cell, American Society of Plant Biologists; School of Biosciences, University of Birmingham, Birmingham, B15 2TT, UK

For all life, the ability to perceive and react to variations in light quality cannot be understated. Mammals achieve this using their eyes, which contain specialized neurons called rod and cone cells, while plants and cyanobacteria possess a suite of sensory photoreceptors that allow them to integrate signals from specific wavelengths of light. The phytochromes (Phys), for instance, are a family of photoreceptors that perceive red and far-red light in the electromagnetic spectrum between 660 and 730 nm. Phys are dimeric proteins comprised of an N-terminal photosensory core module and a C-terminal output module. Photoactivation of Phys by red light induces reversible structural changes that facilitate their nuclear translocation, promote new protein-protein interactions, and alter gene expression. This meta-stable active state (Pfr) can be reverted to the low-energy, inactive state (Pr) by far-red light or thermally by dark reversion (Willige et al. 2024) (see Fig. 1A).

**Figure 1.**
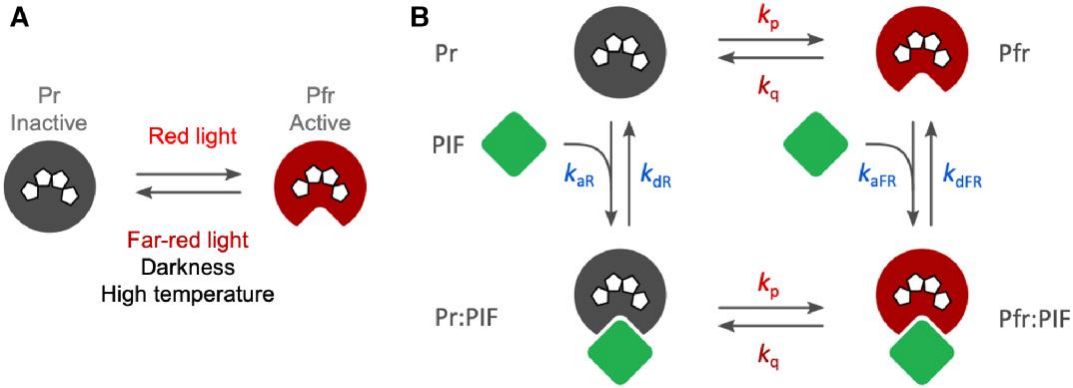
**A)** Schematic describing the reversible Pr⇄Pfr reaction of Phys. While red light activates Pfr formation, far-red light, darkness, and high temperature all promote Pr formation. **B)** Rate constants (*k*) used to model phyB:PIF complex formation. Red light drives the reversible activation of phyB (Pr⇄Pfr; *k*_p_ and *k*_q_) and can occur in or out of complex with PIF. While the rate constants for the bimolecular binding and dissociation of PIF with Pfr are defined as *k*_aFR_ and *k*_dFR_, respectively, the constants for the same reaction between PIF and Pr are defined as *k*_aR_ and *k*_dR_. Adapted from Figure 2 of Yi et al. (2024), copyright American Society of Biologists.

In plants, Phys regulate many aspects of development, including germination, flowering time, shade avoidance, and pathogen resistance. Remarkably, Phys (specifically phyB) were recently implicated to be the primary thermosensor in Arabidopsis (Legris et al. 2016). These authors showed that higher temperatures promote the Pfr > Pr reversion, inactivating PhyB and promoting the activity of its nuclear target proteins, the PHYTOCHROME INTERACTING FACTORS (PIFs). In new work, **Chengwei Yi (**Yi et al. 2024**)** and colleagues serendipitously identify a novel facet to Phy regulation and propose a new model that challenges the dogma surrounding Phy activity under high light and temperature.

Initially, the authors set out to describe the interaction kinetics between Pfr phyB and various PIF-mScarlet fusions at different temperatures using fluorescence resonance energy transfer (FRET) spectroscopy. Acting as a FRET pair, Pfr phyB-PIF binding reduced mScarlet fluorescence, allowing quantification of complex formation under a range of temperatures, wavelengths, and light intensities. As phyB activation is required for PIF interaction, the authors logically expected to observe a stronger interaction (weaker PIF-mScarlet fluorescence) between the two with increasing red light intensity, but remarkably the opposite was true. Under increasing (but physiologically relevant) light intensities, it became clear that the interaction between phyB and several PIFs/PIF subunits grew weaker. To understand this hitherto counterintuitive observation, the authors implemented a mathematical model to understand the rate constants for different features of the phyB:PIF interaction (see Fig. 1B).

The authors proposed that high light intensities accelerate the bi-directional Pr⇄Pfr photoconversion, which becomes so fast that it competes with the rate of phyB:PIF complex formation. To test this experimentally, the authors used an optogenetic circuit in which reporter gene expression would be induced by phyB:PIF complex formation under red light (Müller et al. 2013, 2014). In both mammalian cells and Arabidopsis protoplasts, reporter expression was significantly lower at higher light intensities, confirming their observation that phyB:PIF binding is attenuated by these conditions despite having no influence on the steady-state ratio of Pr:Pfr.

Having explained this unexpected outcome in their initial proof of principle, it was now possible to address their original question: How does temperature influence the interaction between phyB and PIFs? The authors modeled the association/dissociation kinetics for phyB:PIF at different temperatures, which even included adjustments for changes in viscosity of the reaction mixture at different temperatures. Despite higher temperatures promoting an increased rate of Pfr > Pr thermoreversion, the authors calculated that the rate of dissociation increased 6.5-fold from 15 °C to 30 °C degrees, while the rate of association for phyB:PIF increased by only 1.3-fold. This suggests that increased temperatures, much like high light intensity, act to destabilize the phyB:PIF interaction. Furthermore, the authors observed that different PIF proteins (PIF3, PIF6) and their functional domains are subject to differing rates of association/dissociation with phyB, suggesting that the thermoresponse might be directly regulated by temperature-dependent binding affinity.

Since truncated constructs of phyB, which lack all or part of the C-terminal output module, were used for these studies, the authors recognize that their findings will be of more immediate benefit to the optogenetics community. Nonetheless, their observations raise some interesting questions in photobiology. For instance, is complex formation between phyB and its multiple other interactors (e.g. COP1/SPA) also attenuated at high light fluence rates? And if the attenuation of phyB:PIF complex formation under high light is physiologically relevant to its perception in planta, is the reverse true for phyA, which perceives low-light fluence rates? Answers to these questions, for now at least, remain in the dark.

## Dive Curated Terms

The following phenotypic, genotypic, and functional terms are of significance to the work described in this paper:

PIF3 Gramene: AT1G09530
PIF3 Araport: AT1G09530
